# Supplementary material for: Barriers and facilitators for the provision of radiology services in Zimbabwe: A qualitative study based on staff experiences and observations
Source: PLOS Glob Public Health. 2023 Apr 14;3(4):e0001796. doi: 10.1371/journal.pgph.0001796 (PMC10104335; doi:10.1371/journal.pgph.0001796)
Supplement: S1 Appendix — (DOCX) [file pgph.0001796.s001.docx]

Appendix A

**Table A1: Categories of factors affecting radiology services identified from published literature, guidelines and standards (including WHO, the Royal Australian and New Zealand College of Radiologists, International Atomic Energy Agency, European Society of Radiology and the UK Royal College of Radiologists)**

| **Category** | **Details from standards related to category** |
| --- | --- |
| Facilities | The practice facilities are expected to support the delivery of safe, quality diagnostic services. They should be clean and constructed to optimise patients/service users’ comfort, privacy and to accommodate those who require special needs.[14] |
| Equipment | Radiology equipment including software should be appropriate to its use, appropriately maintained such that the acquired imaging results are consistently of high diagnostic quality.[14,15,16] All equipment required for all procedural activities carried out at any institution or centre are available, functional, capable, calibrated and compliant with regulatory requirements. Within the radiology department there should be equipment register of equipment by name and serial numbers including manufacturers’ manuals. Equipment must have an appropriate program of quality control testing and recorded and kept in the department according to medical devices and equipment management policy. [14,16,1] |
| Personnel | Radiographers and Radiologists should be trained and licenced to practice by a regulating board according to the requirements of their country of work. [14,17] Radiology departments should have among other personnel a radiologist and a radiographer. [15] As clinical imaging specialists, the role of radiologists is to provide medical oversight and leadership in imaging, give advice regarding selection of imaging examinations, interpret imaging findings, develop protocols (that include referral criteria) for imaging examinations, ensure appropriate safety protocols are in place for radiation exposure and contrast injections, consult with other clinicians. Radiographers ensure quality diagnostic images are produced as expected, patient safety and other tasks in radiology departments to meet Radiography clinical performance standards.[15,18] |
| Professional supervision | To maintain safety and quality of each patient’s imaging examination professional supervision requirements are important and should be adhered to. Student radiographers must have on site supervision from qualified radiographers at all the times when giving medical radiation to patients.[14,19] |
| Request forms | Justification of imaging request cards is of paramount importance in radiology. A well standardised system of vetting the request for appropriateness must be in place to avoid wrong imaging examination performed and unnecessary medical radiation to patients.[20,21]  Radiology departments must ensure that the following information is provided on request cards prior to an imaging examination being performed: patient name, date of birth, study requested, clinical indication for the examination in detail including relevant clinical history of the patient, the question to be answered, date of request, signature and printed name of requester and their contact details. [14,16,17] |
| Safety | All diagnostic imaging examinations must be conducted in a manner which ensures the safety of patients, personnel and environment. [14,15] The department should monitor and accommodate the environmental conditions as required by relevant imaging specifications and where they influence safety and quality of radiology imaging services.[14] Quality control testing of equipment ensures safety to patient, personnel and environment. All equipment found defective must be taken out of service, clearly labelled and must not return to service until serviced/repaired.[22] Once repaired they must be calibrated and checks to meet relevant accepted criteria usually by medical physicists should be carried out.[14,22]  Radiation safety must be adhered to without fail. The application of ALARA (as low as reasonably achievable) to each radiological procedure perform should be applied all the time and document radiation safety policies and procedure aiming to reduce or minimise radiation exposure.[14,21] Patient radiation doses must be recorded and aggregated annually in order to establish Practice Dose Reference levels (PRLs).[14] Other safety issues are use of contrast media and infection control. In regard to use of contrast media, protocols must be clear for the management of adverse reactions. Effective infection control policies and measure must be in place and adhered to by all staff members in the radiology department. |
