# Supplementary material for: Barriers and facilitators for the provision of radiology services in Zimbabwe: A qualitative study based on staff experiences and observations
Source: PLOS Glob Public Health. 2023 Apr 14;3(4):e0001796. doi: 10.1371/journal.pgph.0001796 (PMC10104335; doi:10.1371/journal.pgph.0001796)
Supplement: S2 Appendix — (DOCX) [file pgph.0001796.s002.docx]

## S2 Appendix B: Interview protocol

**Guiding questions for Focus group discussion and Semi-structured interview**

1. I know you work so hard on daily basis in this hospital. What can you say about the good things you do in brief?
2. What are the problems you face as Radiology department in terms of providing services to the public? Explain please
3. How often does your equipment break down? I mean across all modalities.
4. In terms of time, how long does it take for the equipment to be repaired and what are the challenges you face.
5. In brief can you explain about reliable supplies of clean water, electricity, spare parts and consumables?
6. Let us discuss about radiation protection measures and regulations in medical imaging especially for optimization of radiation delivered during CT and fluoroscopy
7. Do you have enough Radiologists? If not, how does that affect your services?
8. Explain the time frame for a report to be available after medical imaging of patients and do you think in your own words it is appropriate time for report to be available in terms of patient management.
9. Do you have enough staff (Radiographers) and explain please?
10. Can you explain what the impact of these limitations could be to Public Health
11. How do you make sure that the radiographers are developed and prepared to take some of the radiologist duties like what is happening in other countries?
12. Explain in short what you think should be done for optimal radiology services to be offered to service users.
13. Do you have anything to say that will add up to this interview?
