# Supplementary material for: Barriers and facilitators for the provision of radiology services in Zimbabwe: A qualitative study based on staff experiences and observations
Source: PLOS Glob Public Health. 2023 Apr 14;3(4):e0001796. doi: 10.1371/journal.pgph.0001796 (PMC10104335; doi:10.1371/journal.pgph.0001796)
Supplement: Attachment — Submitted filename: Response to reviewers.docx [file pgph.0001796.s005.docx]

4 November 2022

To the Editorial team, PLOS Global Public Health

Dear Sir/Madam,

***Re: Manuscript number PGPH-D-22-00502***

We are pleased to provide a revised manuscript in response to the helpful comments received from the reviewers.

Please find below a point-by-point response to comments from the editor and reviewers. We look forward to hearing the outcome of our revised submission.

Yours sincerely,

Saba Hinrichs-Krapels, on behalf of the authors.

| **Responses to reviewer 1** | |
| --- | --- |
| **Comment** | **Our response** |
| The title: Since the paper deals with experiences and perceptions of radiology human resource, the title “Barriers and facilitators to the provision of radiology services in Zimbabwe: a qualitative study” is too generic and does not explicitly focus on the aim of the study. This study sought out to explore the experiences and perceptions of radiology workers which impact provision of quality radiology services in Zimbabwe. and; a suggested title is “Radiology professionals’ perceptions and experiences affecting delivery radiology services in Zimbabwe” or “Experiences and perceptions of radiology workers that affect provision of quality radiology services in Zimbabwe”. | We thank the reviewer for this suggestion and agree that the title did not reflect the aims of the study explicitly. While reflecting on the reviewers’ further points below, we decided to keep the title closer to its former wording, but instead revised the aims of the study to reflect the study’s two main goals more appropriately. These now read:  “*Our qualitative study aimed to: (a) identify barriers for delivering radiology services, and (b) suggest potential facilitators for improvement of radiology service delivery in the Zimbabwean context. We achieve this by eliciting the perspectives and experiences of radiology staff.”*  In response to a comment raised further below, we also note which method was used to address each of the two aims.  The title therefore is now: “**Barriers and facilitators for the provision of radiology services in Zimbabwe: a qualitative study based on staff experiences and observations**” |
| Justification: There is need for a paragraph to justify the study i.e; Radiology services in LMIC are inadequate to meet the health needs of these countries. Radiology is critical for accurate disease diagnosis, screening and image-guided therapies. Imaging services in low and middle income countries have received little attention. Most research has analyzed physical infrastructure and equipment, however, experiences and perceptions of service providers is critical to effective service provision. This area has not yet been researched on and presents a significant knowledge gap. There is therefore a justification for exploring the experiences and perceptions of radiology workers as they engage in service delivery, and explain how this affects quality service provision. | We thank the reviewer for this suggestion. Our opening two paragraphs are intended to provide this justification, and we have added lines to state this more specifically. Some minor edits have been made to the text in lines 57-70. In addition, we have added the following line and editions in line 72-76:  *“Furthermore, we found no studies to date that specifically sought to identify both barriers and facilitators to providing better radiology service provision from the perspective of radiology staff. Including their perspective allows for a deeper understanding of how such infrastructure and equipment challenges provide a barrier to their work, and what insights and experiences of staff can lead to potential facilitators for improvement.”* |
| An exploratory qualitative study design with triangulation of data sources; observation, interview and focus group discussions is appropriate for investigating experiences and perceptions. Although the researchers imply triangulation Line 141, they don’t explicitly state how triangulation was conducted, which research question was answered by triangulation and the type of triangulation used. The study aimed at achieving rigor through triangulation of methodologies and data sources. Ideally for triangulation to be effective, the different methodologies namely interview, focus group discussions and observations must all address the objectives and explore the same variables. In this case, the variables were experience, perceptions and attitudes. It is apparent that the observation methodology did not focus at experience, perceptions and attitudes. Furthermore, an attempt should be made to ensure the target groups are the same for each method of triangulation. As an example, radiographers should feature in the interviews, FGDs and they should be the target for the observation component. Similarly, radiologists should feature in the interviews, FGDs and they should be the target for the observation component. Managers should also feature in the three methods of data acquisition. The uniform involvement of all types of participants in the three methodologies, in addition to them all addressing the same issues, ensures data homogeneity, comprehensiveness and saturation and allows for comparison of responses and gleaning of data for concepts, subthemes and themes. This study does not ably depict that homogeneity | We thank the reviewer for this valid concern. We are mindful of the different approaches to triangulation available in the literature that guides qualitative research in healthcare settings (e.g. Mays N, Pope C. Assessing quality in qualitative research. Bmj. 2000 Jan 1;320(7226):50-2.; and Denzin NK, Lincoln YS, editors. The Sage handbook of qualitative research. sage; 2011 Apr 27.).  Against this background, we note that  our study took a different approach to triangulation than that proposed by the reviewer. Obtaining full triangulation was not our objective, and this is also extremely difficult to obtain while collecting data in resource-limited settings. Respecting the points made by the reviewer, however, we have clarified the exact purpose of each data collection method and added extra lines to explain the approach we have taken to triangulation, which in our case focusses on validating the data obtained, rather than full triangulation. Through this extra text, we make clear that the observations did not focus on experience, perceptions and attitudes, but focussed on validating the participants’ responses in the other two data collection methods with respect to responses that referred to the context and environment in which staff were delivering services. This text is copied below (lines 177-183):  “*Given our emphasis on seeking the perspective of radiology staff for delivering quality services, our main method for data collection comprised of* ***semi-structured interviews*** *(n=13 participants from a range of roles and hospitals) and three* ***focus groups*** *(n=24 radiographers from the four hospitals). To provide further contextual information on the realities of delivering radiology services, and improve the validity [15] of data collected from the responses from the staff, we conducted four half to full days of* ***field observations*** *at the three public hospitals, and not the private hospital.”* |
| Table 1 should be in an annex or just referred to, but should not form the body of this paper, since it is not a main finding but it is just a reference standard. | We thank the reviewer for this suggestion. Since we created Table 1 by collecting standards from multiple sources, we took the suggestions from the reviewer and included it as an annex instead of in the body of the paper. |
| Lines 117 to 119 are confusing. The numbers don’t tally. Does the sample comprise of a total of 4 (three public and one private) or a total of 6 (four public and two private) hospitals? | We realise the confusion in the previous text. Our intent is to show how our sample of 4 hospitals compares to the total number of available hospitals in the whole region. We have modified this to: “*Data was collected between October and November 2018 in all 3 public (labelled Hospitals A, B and C in Table 1) and 1 private hospital (Hospital D), all in the Harare metropolitan area. This sample represented a significant share of the total number of hospitals in the region, since at the time of our study there were a total of four public and two private hospitals that offered radiology services in the city.”*(now lines 173-177) |
| Why were only 13 staff interviewed? Did they interview until the responses were saturated? How was saturation of the answers ensured? | Our aim was to recruit a sample of respondents with maximum variation within the constraints of the fieldwork, but we were able to observe saturation as well which we have noted in our methods section (lines 216-219) |
| What governed the choice of the carder who participated in the interviews and those who participated in the focus group discussions? Why were the managers, senior radiographers, radiologists and consultant physician not included in the focus group discussions? If as the authors state that the exclusion of higher carder was to remove un-due influence, a focus group discussion composed of these higher carders only should have been conducted. | Indeed, as pointed out by the reviewer, exclusion of the managers, senior radiographers, radiologists and consultant physicians from the focus group allowed a more open discussion and removal of un-due influence, as noted in our manuscript. We agree that a separate focus group comprising only of these senior staff could have added extra depth to the study, and have stated this in the limitations section (lines 656-676). |
| There is an inconsistency in the number of hospitals where observation was conducted. The test states two hospitals yet the table shows three. Why were observations only conducted in two of the facilities and what factors guided the selection of these two facilities? One day is may not be sufficient to conduct an observation study since activities and functions may vary through an entire week and month. The observation based on just one day may therefore be under-representative of the real situation. | As now noted in the re-framed aims of our study, these observations served to validate contextual information given by the interviewees and focus group participants, and not provide extra data and insights. Nevertheless, we do agree that ideally there would have been observations in all four hospitals over a longer period covering different days of the week, and have noted this in the limitations.  We also corrected an error in the text – it now reads that observations were conducted at all three public hospitals, not the private hospital (lines 180-183) |
| The field observations should have focused at the same objectives namely attitude, experiences and perceptions to assess how these were lived out and evident in the day to day practice, instead, the observers focused on physical infrastructure, buildings and equipment. Observations should have targeted staff and their behavior. The information from these observations can therefore not be triangulated in the analysis since the focus is entirely different and not in line with the study aims. | As noted above in response to previous comments from the reviewer, we have re-framed our aims to more accurately depict the goals of our study, and specified in our text that the interviews and focus groups were the main sources of data collection. Therefore, barriers and facilitators were both derived from the perspective of staff via interviews, and focus groups. And the observations served to gather some contextual information, which could include physical infrastructure, and comparison with their reported behaviour. |
| In the results, the researchers need to structure their lay out in relation to their objectives i.e., we need to see results relating to experiences and perceptions. They should also these results by data collection technique as stated in the methodology.  Additionally, some of major findings don’t however fall in the main realm of experiences and perceptions and don’t address all objectives. The authors list 2 objectives namely:(a) describe staff experiences and perceptions in delivering., (b) identify staff barriers for delivering radiology services. In addition, these objectives and results don’t align well with the study title. Question is; are the experiences also the facilitators and barriers? There is need for clarity of what the researchers set out to do.  The poor basic infrastructure, lack of equipment, and consumables, suboptimal equipment maintenance; and integration of radiology services into other hospital functions are neither an experiences or a perceptions and don’t have a direct linkage to staff. They could have been reported on but they should not be at the forefront of the findings and discussion since the paper deals with radiology workers and their “experiences and perceptions”. | We thank the reviewer with this suggestion. As noted above, we have re-framed the aims of our study to make it much clearer that our goal was to gather barriers and facilitators to delivering services. The ‘perceptions and experiences’ were deleted as these were the methods through which we obtained our goal, i.e. from the perspective of staff.  Given these re-framed aims of our study (to report on barriers and facilitators), we feel the current structure of the results better conveys the overall findings, since all data collection methods contributed to identifying barriers and facilitators.  We feel it is now clear that the major findings are the barriers (the first of which is a physical infrastructure barrier to delivering services, and others relating to management and support), which we report in results (i)-(iv). The facilitators were elicited by studying the transcripts and analysing the motivations of staff in the interviews and focus groups, which were summarised in result number (v). |
| The “strong sense of motivation” would fall within the subject of experiences and perception and should have been the main area of discussion. It is not mentioned however that this motivation was universal across all carders and facilities and whether there are some facilities or carders who were not strongly motivated. The explanation to this strong sense of motivation is also not given. | We thank the reviewer for pointing out this omission regarding whether or not this motivation was universal across all carders and facilities. We have clarified that staff from all hospitals demonstrated this sense of duty and motivation (now lines 502-506).  We could not discern from the data why this strong sense of motivation existed, so we do not feel it would be correct to report on the explanation for this. We agree it is an interesting point and can be researched further, which we now state in the discussion (line 543-547) |
| The shortage of radiology staff and skills development justifiably falls within the realm of the research but there is need to explore and get the views of the staff on the optimal number of professionals and their ranks and areas of specialization, plus the anticipated areas of skills training. | We thank the reviewer for this comment. Further research would be needed to address this important question. Participants in our study did refer to some numbers that suggest what this optimum number could be, but we felt that these numbers cannot be reported based on perceptions and views of staff. We report only on their perceptions of having shortages as an indicator of the limited resources for the patient loads they experience. |
| The authors should reorganize the results and discussion to focus more at the experiences, perception and attitude which impact radiology service provision, rather than the physical aspects of infrastructure and equipment since these are covered in other publications (as indicated in the introduction), and the justification of this research as well as the objects were to explore how these experiences, perception and attitude impact radiology services. The authors may then propose ways of mitigating the impact of these on service provision. | As noted to the responses given to the reviewer above, we have re-framed the aims and felt that the current structure in the results more adequately reports on the themes under our two main aims: barriers and facilitators, which were gathered from all data sources. |
| The researchers focus not on their study results in their discussion. They need to discussion why their results turned out the way they did and compare with the existing body of knowledge. The discussion section should also be structured according to the results based on the study objectives. See line 463 | We are particularly grateful to the reviewer for this helpful comment, and agree we needed to make our discussion relate more closely both to our aims and our results. We have now renamed the two discussion sections, the first to focus on the barriers (relating our findings back to the literature), and the second on what we view as facilitators for improvement (and our justification and arguments for doing so). We feel this re-framed discussion more strongly makes the arguments and final thesis in our article, and are very grateful to the reviewer for identifying this for us. |
| Conclusion We don’t see any conclusions relating to the perceptions. | We thank the reviewer for this final comment. With our re-stated aims focussing on barriers and facilitators, we feel that the conclusion now adequately summarises our findings in these two respects. Further, we emphasised that the sense of motivation and investments in staff is one of the underlying facilitators that we view for making improvements in radiology service delivery (lines 623-636) |
| **Responses to reviewer 2** | |
| A good study whose findings add to a knowledge gap, and point to key issues within the radiology services provision in the country, and may stimulate relevant actions towards the infrastructural, equipment, skills and services required to optimize the services. | We thank the reviewer for these encouraging comments. |
| The title is appropriate for the study aims, methodology and conclusions. The statement of intent is clear, the methods are well articulated with use of validation and benchmarking to bridge the local gaps, and this makes for a comprehensive picture. | We thank the reviewer for this feedback. |
| Though qualitative the authors needed to include some background quantitative data on workload statistics and specifics on burden of disease for which radiology is required in order to adequately put into perspective the deficit in services provision. | We thank the reviewer for this important suggestion. We have made a few changes to respond to this feedback. First, we moved some of the text that previously was included in ‘Methods/settings’ to the Introduction section. This is intended to set the Zimbabwean context at the start of the article, so that the results and discussion can be discussed in this light. Second, we added extra statistics from the OECD on healthcare expenditure, and any existing workload statistics and deficits in radiology services to provide further context for the region. This is now in lines 90-113. |
| The lack of input from the respective hospitals managers, the radiation protection authority, and Ministry of Health who have the capacity to act on the findings should be addressed. | We thank the reviewer for this feedback and realise the input from these individuals would help galvanise the impact of our study. However, the study’s intention is to focus on those who deliver the services on the ground, and we have reported the findings to be limited to these individuals. To respect this point raised by the reviewer, we do include a line to highlight the importance of including wider stakeholders for following up the results of this study. (lines 661-671) |
| At least for the hospitals A to D the key findings should have been tabled for specific improvements, and clearer comparisons drawn. | We thank the reviewer for this suggestion. We did consider presenting the results in this structured way. However, given the small sample number, we felt that specific demarcation of the findings according to each hospital may compromise anonymity of the findings. We therefore opted to include general themes of practices across the hospitals to present problems and challenges generally. |
| A limitation is lack of data from the second city, Bulawayo, and other cities which offer radiology services. This further limits the conclusions on the specifics of equitable access to the services and what spectrum of services is available in other locations in the country. The authors must therefore declare the latter as a weakness of their study to open for further study. | We thank the reviewer for this comment, and agree this is a limitation of our study. We have added a line under limitations to declare this limitation (656-657) |
| The authors point to the challenges in equipment purchase and they should make sound recommendation from their findings, such as service contracts which take into account serviceability by the equipment suppliers | We thank the reviewer for this comment. We agree that wider organisational changes (including procurement approaches) should have been included as part of the discussion, and we now include this in lines 638-647). |
| **Responses to editor** |  |
| When revising your manuscript, please address reviewer 1's comments in terms of clarifying the study's justification as well as the methods. As part of these clarifications, it would be helpful to go into some more detail about the Zimbabwean healthcare system, as mentioned by reviewer 2.  We would also ask that you reframe the work's findings more clearly, as well as its conclusions. | Thank you for this clear guidance. In our response primarily to reviewer 1, we have re-framed the aims of the study and feel these more accurately depict the overarching goal. We also report above how this matches the way we have structured the findings, and final conclusions. We hope these modifications response adequately to the concerns raised by the reviewers in framing the work’s findings, as well as situating its conclusions in light of our aims.  Finally, we have revised the section in the introduction to provide more detail on the Zimbabwean healthcare system, to further provide context and implications of our study. |
